# Triggered Golgi membrane enrichment promotes PtdIns(4,5)P2 generation for plasma membrane repair

**DOI:** 10.1083/jcb.202303017

**Published:** 2023-05-09

**Authors:** Xinan Meng, Chandra Sugiarto Wijaya, Qingfang Shao, Suhong Xu

**Affiliations:** 1 International Biomedicine-X Research Center of the Second Affiliated Hospital, Zhejiang University School of Medicine and the Zhejiang University-University of Edinburgh Institute, Haining Zhejiang, China; 2 Department of Burn and Wound Repair of the Second Affiliated Hospital, Center for Stem Cell and Regenerative Medicine, Zhejiang University School of Medicine, Hangzhou Zhejiang, China; 3 School of Basic Medical Sciences, Zhejiang University School of Medicine, Hangzhou Zhejiang, China

## Abstract

The maintenance of plasma membrane integrity and a capacity for efficiently repairing damaged membranes are essential for cell survival. Large-scale wounding depletes various membrane components at the wound sites, including phosphatidylinositols, yet little is known about how phosphatidylinositols are generated after depletion. Here, working with our in vivo *C. elegans* epidermal cell wounding model, we discovered phosphatidylinositol 4-phosphate (PtdIns4*P*) accumulation and local phosphatidylinositol 4,5-bisphosphate [PtdIns(4,5)*P*_2_] generation at the wound site. We found that PtdIns(4,5)*P*_2_ generation depends on the delivery of PtdIns4*P*, PI4K, and PI4P 5-kinase PPK-1. In addition, we show that wounding triggers enrichment of the Golgi membrane to the wound site, and that is required for membrane repair. Moreover, genetic and pharmacological inhibitor experiments support that the Golgi membrane provides the PtdIns4*P* for PtdIns(4,5)*P*_2_ generation at the wounds. Our findings demonstrate how the Golgi apparatus facilitates membrane repair in response to wounding and offers a valuable perspective on cellular survival mechanisms upon mechanical stress in a physiological context.

## Teaser

Meng et al. report that upon plasma membrane damage in a cell, the Golgi apparatus accumulates at the wound site and facilitates membrane repair by converting PtdIns4P to PtdIns(4,5)P2 in *C. elegans*. This finding advances our understanding of cellular survival mechanisms under mechanical stress.

## Graphical Abstract

**Figure:**
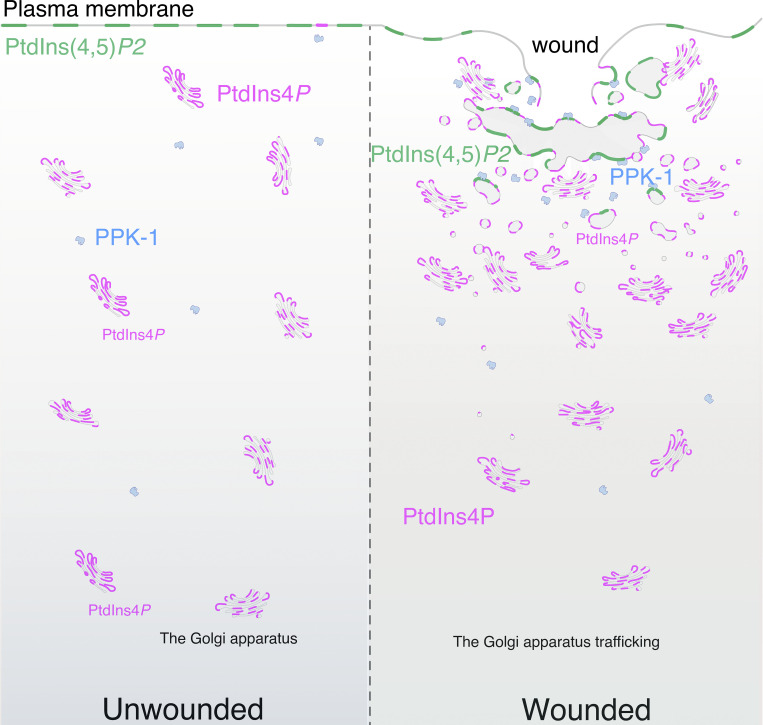

## Introduction

The plasma membrane acts as a cellular barrier to defend against extracellular hazards that threaten cell survival. The loss of plasma membrane integrity has been causally linked to inflammation, muscular dystrophies, aging, and neurodegenerative diseases (Ammendolia et al., 2021). Upon plasma membrane damage, cells can quickly sense the damage and then activate repair program(s) to remodel the membrane structure to preserve the membrane integrity (Demonbreun et al., 2016; Tang and Marshall, 2017). Cells have evolved efficient pathways for repairing membrane damage, including, for example, the use of Ca^2+^-dependent fusion of vesicular patches (Davenport et al., 2016; Terasaki et al., 1997); the removal of damaged membranes via endocytosis (Idone et al., 2008); ESCRT III-mediated membrane shedding (Jimenez et al., 2014; Scheffer et al., 2014); and membrane fusion to reseal the damage (Cai et al., 2009; Tam et al., 2010). Despite these mechanisms, large-scale damage to the plasma membrane depletes various membrane components, leading to a loss of membrane function (Terasaki et al., 1997). However, it is not yet understood how these components are locally generated to restore the function of the plasma membrane.

Phosphatidylinositol 4,5-bisphosphate (PtdIns(4,5)*P*_2_) is recognized as a crucial lipid component of the plasma membrane that organizes cell shape (Posor et al., 2022). It maintains membrane structural integrity by regulating the actin cytoskeleton and is indispensable for the processes such as clathrin-coated vesicle endocytosis, budding, signal transduction, membrane trafficking, and many other cellular activities (Mandal, 2020; Posor et al., 2022). Disruptions in the levels of PtdIns(4,5)P2 or its regulation have been linked to a number of human diseases, including cancer, neurological disorders, and immunological disorders (Mandal, 2020). PtdIns(4,5)*P*_2_ is converted by PtdIns4*P* 5-kinase (PI4P5K) from PtdIns4*P*, which is primarily located on the membrane of the Golgi apparatus (Glick and Nakano, 2009). Nevertheless, the mechanisms through which PtdIns(4,5)*P*_2_ is regenerated after depletion (for example, upon cell wounding) and the potential involvement of the Golgi apparatus in repairing damaged membranes remain unclear.

Here we report that wounding triggers PtdIns4*P* delivery and PtdIns(4,5)*P*_2_ generation at the wounded membrane in *Caenorhabditis elegans* epidermal cell. PtdIns(4,5)*P*_2_ generation at the wound site depends on PtdIns4*P*, PI4K, and PI4P5K PPK-1. We further discovered that wounding triggers the enrichment of the Golgi membrane to the wound site to provide PtdIns4*P*, which serves as a substrate for PtdIns(4,5)*P*_2_ generation during the membrane repair.

## Results and discussion

### Wounding triggers PtdIns4P delivery and PtdIns(4,5)P2 generation at the damaged membrane

**Figure 1.**
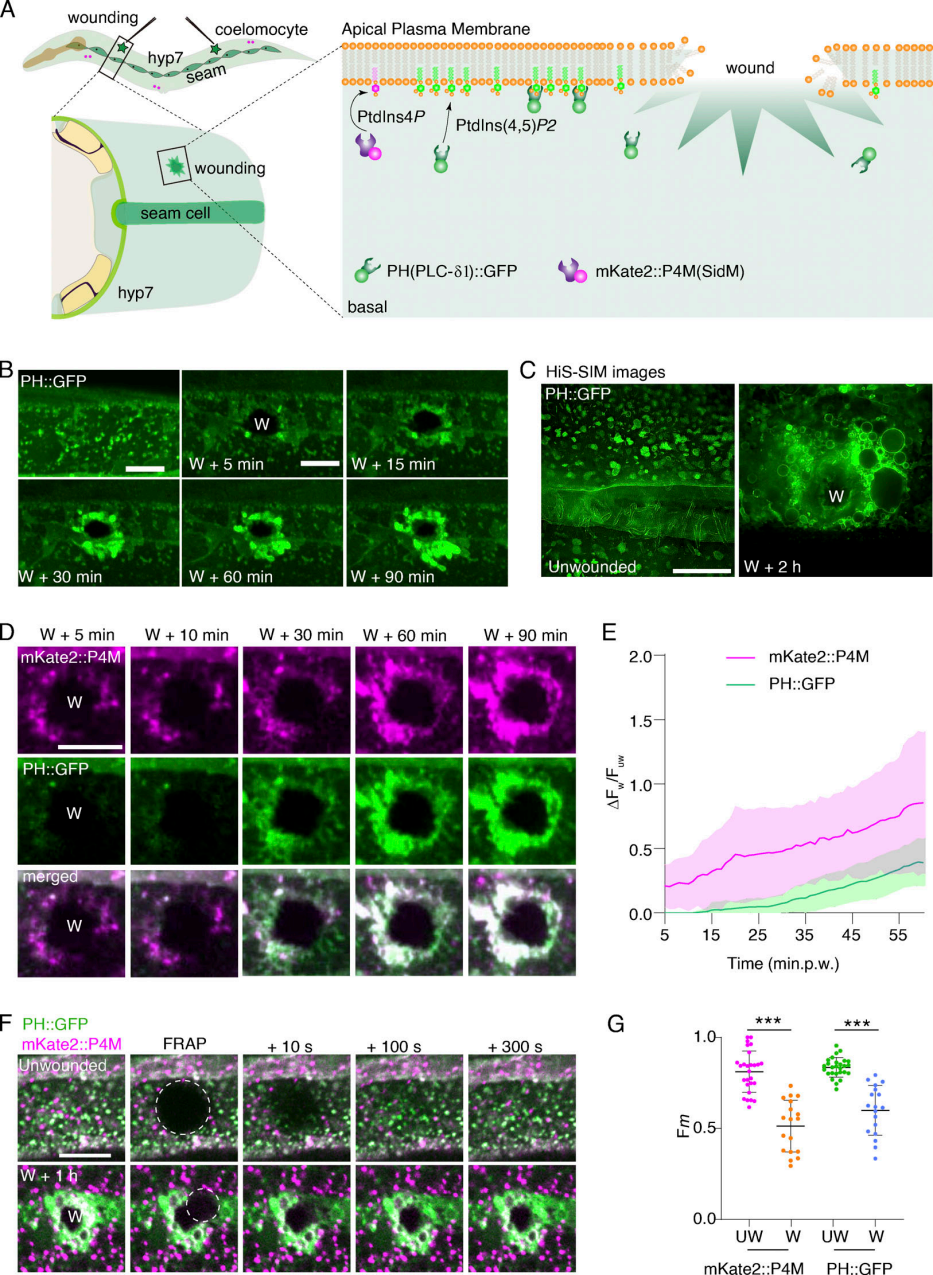
**Wounding triggers PtdIns4*P* accumulation and PtdIns(4,5)*P***_**2**_ **generation at the wound site. (A)** Illustration showing *C. elegans* epidermal cell hyp7 as a model for membrane repair. In this study, membrane components PtdIns(4,5)*P*2 were labeled by the PH domain from PLC-δ1, and PtdIns4*P* were labeled by the P4M domain from *L. pneumophila* SidM. **(B)** Time-lapse images showing the accumulation of PH::GFP at the wound site. “W” indicates the wound site. *Pcol-19-PH::GFP (zjuSi175)* transgenic animals were used for wounding and imaging. Scale bar: 10 µm. **(C)** Representative HiS-SiM single-plane images of PH::GFP before and after wounding. *Pcol-19-*PH::GFP*(zjuSi175)* transgenic animals were used for wounding and imaging. Scale bar: 10 µm. **(D)** Representative confocal time-lapse images of the recruitment of mKate2::P4M and PH::GFP between 5 to 90 min after wounding. *Pcol-19-*PH::GFP*(zjuSi175);Pcol-19-mKate2::P4M (zjuSi333*) transgenic animals were used for wounding and imaging. Scale bar: 10 µm. **(E)** Line graph showing the intensity change (ΔF_w_/F_uw_) of mKate2::P4M and PH::GFP at different time points after wounding. The plot indicates the mean ± SD (*n* = 6 for mKate2::P4M curve and 8 for PH::GFP curve) at each time point. **(F)** Fluorescent recovery after photobleaching (FRAP) analysis of the recovery capability of mKate2::P4M and PH::GFP before and 1 h after wounding. *Pcol-19-PH::GFP(zjuSi175);Pcol-19::mKate2::P4M(zjuSi333)* transgenic animals were used for wounding and imaging. Scale bar: 10 µm. **(G)** Mobile fraction (F_m_) analysis of FRAP of mKate2::P4M and PH::GFP in F. Error bars represent the mean value ± SD (*n* = 27, 18, 27, 18 from the left to the right), Mann–Whitney test, ***P < 0.001. See also Fig. S1; and Videos 1, 2, 3, 4, 5, 6, and 7.

**Figure S1.**
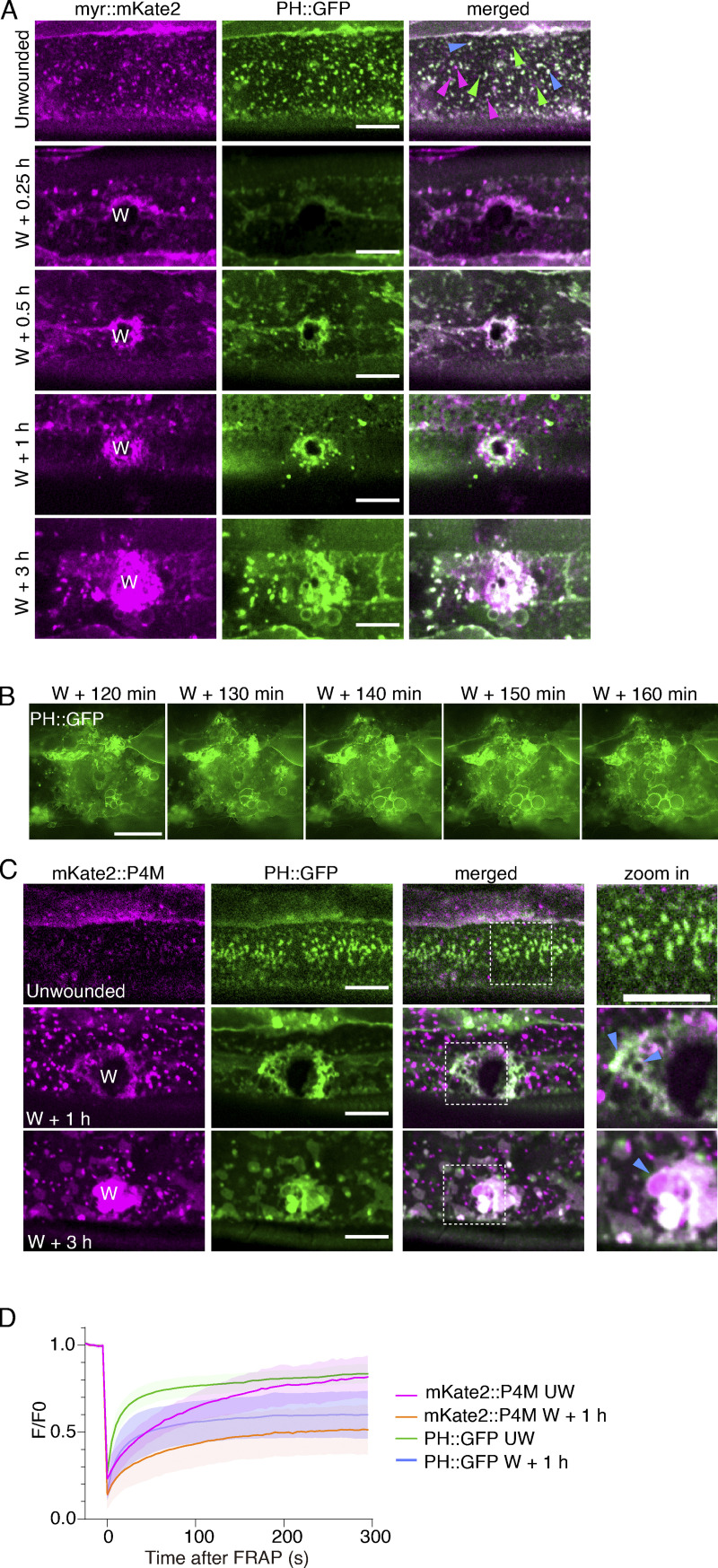
**Epidermal membrane wounding triggers PtdIns4*P* accumulation and PtdIns(4,5)*P***_**2**_ **generation at the wound site, related to** Fig. 1**. (A)** Representative single-plane confocal images showing the colocalization of myr::mKate2 and PH::GFP before and after wounding. Pcol-*19*-myr::mKate2(*zjuSi46)*; P*col-19-*PH::GFP(*zjuSi175*) transgenic animals were used for wounding and imaging. “W” indicates the wound site. White dotted boxes indicate the zoom-in region. Blue arrows pointed to the colocalized puncta, green arrows pointed to PH::GFP puncta, and magenta arrows pointed to myr::mKate2 puncta. Scale bar: 10 µm. **(B)** HiS-SiM time-lapse images of PH::GFP in wounded P*col-19-*PH::GFP(*zjuSi175*) animals. Scale bar: 10 µm. **(C)** Representative single-plane confocal images showing the colocalization of mKate2::P4M and PH::GFP before and after wounding. P*col-19-*PH::GFP(*zjuSi175*); P*col-19*-mKate2::P4M(*zjuSi333*) transgenic animals were used for wounding and imaging. White dotted boxes indicate the zoom-in region. Magenta arrows indicate the colocalized puncta. Scale bar: 10 µm. **(D)** Line graph showing the intensity change (ΔF_w_/F_uw_) of unwounded and wounded worms after FRAP. The mean ± SD at each time point is indicated (*n* = 27, 18, 27, 18 from the top to the bottom).

We decided to investigate the role of the Golgi apparatus in generating PtdIns(4,5)*P*_2_ using our previously described in vivo model for repairing large membrane wounds (Meng et al., 2020; Wijaya et al., 2020). For this, we fused the Pleckstrin Homology (PH) domain of PLC-δ1 with a GFP (Vaughan et al., 2014) to label PtdIns(4,5)*P*_2_ in *C. elegans* syncytium epidermal cell hyp7 under the control of the *col-19* promoter (Fig. 1 A; Wang et al., 2022). Spinning disk confocal microscopy was then used to observe the fluorescent signal of PH::GFP before and after wounding. PH::GFP formed puncta at the plasma membrane with relatively stable and almost colocalized the plasma membrane marker myr::mKate2 (myristoyl signal sequence; Meng et al., 2020; Fig. S1 A and Video 1). We then performed physical punctures using a microinjection needle to the anterior and posterior regions of the hyp7 (Fig. 1 A). PH::GFP signals were temporarily depleted following needle wounding and then slowly recovered at the wound periphery in a matter of minutes (Fig. 1 B and Video 2), consistent with previous observations (Meng et al., 2020; Taffoni et al., 2020; Vaughan et al., 2014). The PH::GFP signals were then observed to increase in fluorescence intensity, eventually covering the entire wound area (Fig. 1 B and Fig. S1 A; and Video 2). The PH::GFP and myr::mKate2 were observed to simultaneously increase at the wound periphery from 15 min to 3 h after wounding (Fig. S1 A and Video 3). Moreover, the locally accumulated PH:::GFP was found to be nearly entirely colocalized with increased myr::mKate2 (Fig. S1 A and Video 3), indicating that the generation of PtdIns(4,5)*P*_2_ was directly linked to plasma membrane repair. Additionally, we did not observe apparent recruitment of PH::GFP puncta from the neighboring region, but instead, a local accumulation of PH::GFP (Video 2). Together, these findings suggest that PtdIns(4,5)*P*_2_ was locally generated at the wound site.

**Video 1.** **Representative single plane confocal time-lapse images showing PH::GFP signal in *C. elegans* epidermal cell before wounding.** Time-lapse was taken for 10 min at 30 s interval. Frame rate: 2 frames per second. Pcol-19-PH::GFP(zjuSi175) transgenic animals were used for imaging. Scale bar: 10 µm. Related to Fig. 1.

**Video 2.** **Representative single plane confocal time-lapse images showing recruitment of PH::GFP to the wound site after needle wounding.** Time-lapse was taken for 90 min at 30 s interval. Frame rate: 10 frames per second. Pcol-19-PH::GFP(zjuSi175) transgenic animals were used for wounding and imaging. Scale bar: 10 µm. Related to Fig. 1.

**Video 3.** **Representative single plane confocal time-lapse images showing recruitment of PH::GFP and myr::mKate2 to the wound site after needle wounding.** Time-lapse was taken for 90 min at 30 s interval. Frame rate: 10 frames per second. Pcol-19-myr::mKate2(zjuSi46); Pcol-19-PH::GFP(zjuSi175) transgenic animals were used for wounding and imaging. Scale bar: 10 µm. Related to Fig. 1.

To further ascertain the local generation of PtdIns(4,5)*P*_2_ at the wound site, we performed improved highly intelligent and sensitive-structured illumination microscopy (HIS-SIM; Zhao et al., 2022) on animals expressing PH::GFP before and after wounding. Consistent with the confocal images, the high-resolution images showed that PH::GFP formed punctated microdomains along the plasma membrane (Fig. 1 C). After needle wounding, PH::GFP coalesced multiple-shaped large vesicle structures that accumulated at the wound site (Fig. 1 C and Fig. S1 B). Over time, the PH::GFP vesicle-like structures enlarged and eventually covered the wounds within hours after wounding (Fig. 1 C and Fig. S1 B; and Video 4). Notably, we observed that the PH::GFP signals were gradually increased locally but not from the neighboring region after wounding (Video 4), reinforcing the notion that the PtdIns(4,5)*P*_2_ is generated locally at the wound site.

**Video 4.** **Representative single plane His-SIM time-lapse shows the recruitment of PH::GFP after needle wounding.** Time-lapse was taken for 48 min at 30 s interval. Frame rate: 10 frames per second. Scale bar: 10 µm. Pcol-19-PH::GFP(zjuSi175) transgenic animals were used for wounding and imaging. Related to Fig. 1.

PtdIns(4,5)*P*_2_ is converted from phosphatidylinositol 4-phosphate (PtdIns4*P*), which is synthesized directly from phosphatidylinositol (PtdIns) by phosphorylation with PI4-kinase (PI4K; Rameh et al., 1997). We then determined whether PtdIns4*P* contributes to PtdIns(4,5)*P*_*2*_ generation in membrane repair. To visualize PtdIns4*P* in *C. elegans* hyp7, we labeled PtdIns4*P* by fusing the P4M domain of *Legionella pneumophila* SidM with the red fluorescent protein mKate2 (Hammond et al., 2014; Fig. 1 A). The mKate2::P4M signal was primarily observed in the cytosol and was not colocalized with PH::GFP at the membrane in the hyp7 (Fig. S1 C and Video 5). Following needle wounding, the mKate2::P4M signal was enriched to the wound periphery earlier compared to PH::GFP, which increased later (Fig. 1, D and E; Fig. S1 C; and Video 6). Unlike the local accumulation of PH::GFP, mKate2::P4M showed relatively dynamic movement at the wound periphery (Video 7). To assess the stability of the accumulated mKate2::P4M and PH::GFP, we performed fluorescence recovery after photobleaching (FRAP) analysis. Neither mKate2::P4M nor PH::GFP at the wound periphery showed efficient recovery to levels comparable to the unwounded region (Fig. 1, F and G; and Fig. S1 D), indicating that the accumulation of PtdIns4*P* and PtdIns(4,5)*P*_2_ at the wound site was not due to the lateral diffusion from the unwounded area.

**Video 5.** **Representative single plane confocal time-lapse images showing mKate2::P4M signal in unwounded *C. elegans* epidermal cell.** Time-lapse was taken for 10 min at 30 s interval. Frame rate: 7 frames per second. Pcol-19-mKate2::P4M(zjuSi333) transgenic animals were used for imaging. Scale bar: 10 µm. Related to Fig. 1.

**Video 6.** **Representative single plane confocal time-lapse images showing recruitment of PH::GFP and mKate2::P4M after needle wounding.** Time-lapse was taken for 90 min at 60 s interval. Frame rate: 10 frames per second. Pcol-19-PH::GFP(zjuSi175); Pcol-19-mKate2::P4M(zjuSi333) transgenic animals were used for wounding and imaging. Scale bar: 10 µm. Related to Fig. 1.

**Video 7.** **Representative single plane confocal time-lapse images showing recruitment of PH::GFP and mKate2::P4M after needle wounding.** Time-lapse was taken for 60 min at 60 s interval. Frame rate: 5 frames per second. Pcol-19-PH::GFP(zjuSi175); Pcol-19-mKate2::P4M(zjuSi333) transgenic animals were used for wounding and imaging. Scale bar: 10 µm. Related to Fig. 1.

### PtdIns(4,5)P2 generation at the wound site depends on PtdIns4P, PI4K, and PI4P5K

**Figure 2.**
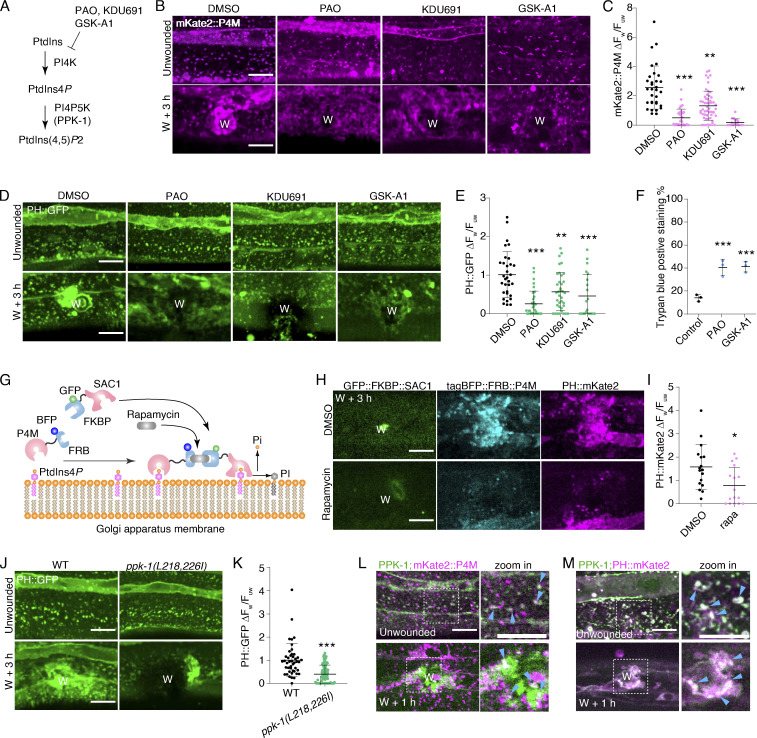
**PtdIns(4,5)*P***_**2**_ **local generation depends on PtdIns4*P*, PI4K, and PI4P5K. (A)** A diagram illustrating the synthesis of PtdIns(4,5)*P*_2_ from PI and PtdIns4*P* by PI4K and PI4*P*5K. Phenyl arsine oxide (PAO), KDU691, and GSK-A1 are inhibitors of PI4K. PPK-1 is the *C. elegans* homolog of mammalian PI4P-5 kinase. **(B)** Representative confocal images showing the effects of PAO (100 μM), KDU691 (500 μM), and GSK-A1 (100 μM) on mKate2::P4M expression before and after wounding. *Pcol-19-mKate2::P4M(zjuSi333)* transgenic animals were used for the experiment. “W” indicates the wound site. Scale bar: 10 µm. **(C)** Quantitation analysis of the mKate2::P4M intensity ratio (ΔF_w_/F_uw_) at 3 h after wounding in animals treated with PAO, KDU691, and GSK-A1 (B). The error bars represent the mean value. ± SD (*n* = 32, 38, 54, 18 from the left to the right), Mann–Whitney test, ***P < 0.001. **(D)** Representative confocal images showing the effects of PAO (100 μM), KDU691 (500 μM), and GSK-A1 (100 μM) on PH::GFP expression before and after wounding. *Pcol-19-*PH::GFP(*zjuSi175*) transgenic animals were used for the experiments. Scale bar: 10 µm. **(E)** Quantitation analysis of the PH::GFP intensity ratio (ΔF_w_/F_uw_) at 3 h after wounding in animals treated with PAO, KDU691, and GSK-A1 (D). The error bars represent the mean value. ± SD (*n* = 33, 36, 37, 21 from the left to the right), Mann–Whitney test, ***P < 0.001. **(F)** Quantitation analysis of trypan blue staining % in N2 worms 6 h after wounding and treatment with PAO (100 μM) and GSK-A1 (100 μM). The error bars represent the mean value. ± SD (*n* = 3), Student’s *t* test, ***P < 0.001. **(G)** An illustration shows the mechanism of the rapamycin-induced FRB-FKBP dimerization system to deplete PtdIns4P. **(H)** Representative confocal images of GFP::FKBP::SAC1, tagBFP::FRB::P4M, and PH::mKate2 after rapamycin (100 μM) treatment on the accumulation of tagBFP::FRB::P4M and PH::mKate2 at 3 h after wounding. P*col-19*-PH::mKate2(*zjuSi321*) II; P*col-19*-P4M::FRB::tagBFP; P*col-19*-GFP::FKBP::SAC1(*zjuEx2307*) transgenic animals were used for wounding and imaging. Scale bar: 10 µm. **(I)** Quantitation analysis of the mKate2::P4M intensity ratio (ΔF_w_/F_uw_) at 3 h after wounding on rapamycin-treated worms (H). The error bars represent the mean value ± SD (*n* = 17 and 16). Mann–Whitney test, *P < 0.05. **(J)** Representative confocal images showing the PH::GFP on *ppk-1(L218,226I)* mutant worms before and after wounding. The leucines at positions 218 and 226 in the PPK-1 protein have been mutated to isoleucine in *ppk-1(syb6134)* mutant animals. Scale bar: 10 µm. **(K)** Quantitation analysis of the intensity change (ΔF_w_/F_uw_) of PH::GFP after wounding (J). Error bars represent the mean value ± SD (*n* = 44 and 115). Mann–Whitney test, ***P < 0.001. **(L)** Representative confocal single-plane images showing the colocalization GFP::PPK-1 and mKate2::P4M before and after wounding. P*col-19*-mKate2::P4M(*zjuSi333*); P*col-19*-GFP::PPK-1(*zjuSi367*) transgenic animals were used for wounding and imaging. The white dotted box indicates the zoom-in region. Light blue arrows indicate the colocalized puncta. Scale bar: 10 µm. **(M)** Representative confocal single-plane images showing the colocalization GFP::PPK-1 and mKate2::P4M, as well as GFP::PPK-1 and PH::mKate2, before and after wounding. P*col-19*-PH::mKate2(*zjuSi321*); P*col-19*-GFP::PPK-1(*zjuSi367*), P*col-19*-mKate2::P4M(*zjuSi333*); P*col-19*-GFP::PPK-1(*zjuSi367*) transgenic animals were used for wounding and imaging. The white dotted box indicates the zoom-in region. Light blue arrows indicate the colocalized puncta. Scale bar: 10 µm. See also Fig. S2 and Video 8.

**Figure S2.**
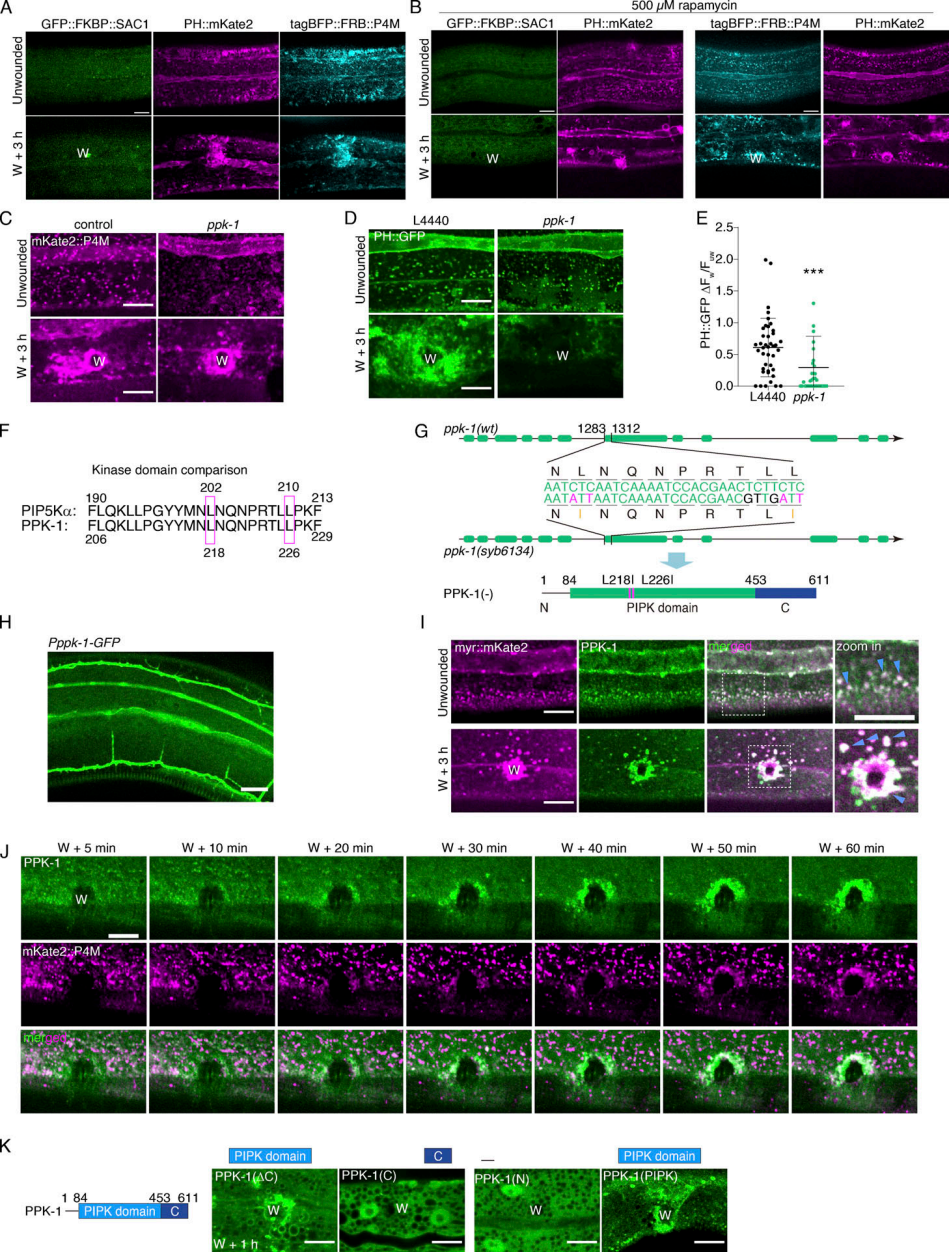
**PtdIns(4,5)*P***_**2**_ **generation depends on PtdIns4*P*, PI4K, and PPK-1 activity, related to** Fig. 2**. (A)** Representative single-plane confocal images showing GFP::FKBP::SAC-1, PH::mKate2, and tagBFP::FRB::P4M without rapamycin treatment before and after wounding. P*col-19*-PH::mKate2(*zjuSi321*) II; P*col-19*-P4M::FKBP12(mTOR)::tagBFP; P*col-19*-GFP::FKBP1A(FKBP 2-108AA)::SAC1(2-517AA)(*zjuEx1859*) transgenic animals were used for wounding and imaging. “W” indicates the wound site. Scale bar: 10 µm. **(B)** Representative single-plane confocal images showing GFP::FKBP::SAC-1, and PH::mKate2 with FRB, as well as tagBFP::FRB::P4M and PH::mKate2 without FKBP before and after wounding. Worms are treated with rapamycin 100 µM). P*col-19*-PH::mKate2(*zjuSi321*) II; P*col-19*-GFP::FKBP1A(FKBP 2-108AA)::SAC1(2-517AA)(*zjuEx1865*) and P*col-19-*PH::mKate2(*zjuSi321)*; P*col-19-*GFP::FKBP1A(FKBP 2-108AA)::SAC1(2-517AA)(*zjuEx1865)* transgenic animals were used for wounding and imaging. Scale bar: 10 µm. **(C)** Representative confocal images of mKate2::P4M before and after wounding with *ppk-1* RNAi treatment. P*col-19*-mKate2::P4M(*zjuSi333*) animals were used for wounding and imaging. Scale bar: 10 µm. **(D)** Representative confocal images of PH::GFP before and after wounding with *ppk-1* RNAi treatment. P*col-19*-PH::GFP(*zjuSi175*) transgenic animals were used for wounding and imaging. Scale bar: 10 µm. **(E)** Quantitation analysis of the PH::GFP intensity ratio (ΔF_w_/F_uw_) at 3 h after wounding (D). The error bars represent the mean value ± SD (*n* = 41 and 33), one-way ANOVA multiple comparisons test, ***P < 0.001. **(F)** Comparison of the amino acid sequence of human PIP5K1A and *C. elegans* PPK-1. **(G)** Experimental design of *ppk-1*(*syb6134*) double point mutation based on the comparison of the nucleotide sequence of *ppk-1*(wt) and *ppk-1*(*syb6134*). Below is the secondary structure of *ppk(syb6134)* mutation. **(H)** Representative confocal images of *Pppk-1::GFP* showing the expression of *ppk-1* in multiple tissues of young adult *C. elegans*. **(I)** Representative single-plane confocal images of myr::mKate2 and GFP::PPK-1 colocalization before and after wounding. P*col-19-*myr::mKate2(*zjuSi46)*; P*col-19*-GFP::PPK-1(*zjuSi367)* transgenic animals were used for wounding and imaging. White dotted boxes indicate the zoom-in region. Magenta arrows indicate the colocalized puncta. Scale bar: 10 µm. **(J)** Representative time-lapse confocal images of the recruitment of GFP::PPK-1 and mKate2::P4M between 5 to 60 min after wounding. P*col-19*-GFP::PPK-1(*zjuSi367);* P*col-19*-mKate2::P4M(*zjuSi333*) transgenic animals were used for wounding and imaging. Scale bar: 10 µm. **(K)** Diagram of PPK-1 secondary structure and representative confocal images of the recruitment of truncated PPK-1 to the wound site. P*col-19*-PPK-1(1–453)::GFP(*zjuEx2147*), P*col-19*-GFP::PPK-1(c)(*zjuEx2142*), *Pcol-19-*PPK-1(1–85)::GFP(*zjuEx2265,) Pcol-19-*PPK-1(84–457)::GFP(*zjuEx2267)* transgenic animals were used for wounding and imaging. Scale bar: 10 µm.

To determine whether the PtdIns4*P* contributes to the generation of PtdIns(4,5)*P*_2_ in membrane repair, we treated animals with Phenylarsine oxide (PAO) and KDU691, inhibitors of PI4K (McPhail and Burke, 2020; Wiedemann et al., 1996), to reduce the level of endogenous PtdIns4*P* (Fig. 2 A). Both drug treatments inhibited the increased signal of mKate2::P4M (Fig. 2, B and C) and PH::GFP at the wound site compared to the controls (Fig. S2, D and E). Additionally, we treated animals with GSK-A1, which specifically inhibits the activity of the PI4Kα (Bojjireddy et al., 2014; Fig. 2 A), and observed that both mKate2::P4M and PH::GFP signals were not increased at the wound site (Fig. 2, B–E). Furthermore, PAO and GSK-A1 considerably increased the positive staining ratio of Trypan Blue (TryB; Fig. 2 F), which is a membrane-impermeable dye used in the previous study that only penetrates damaged membranes (Meng et al., 2020), suggesting that delivery of PtdIns4*P* at the wound site contributes to the membrane repair. Together, these pharmacological inhibitor experiments suggest that PtdIns(4,5)*P*_2_ generation depends on the PI4K activity and the accumulation of PtdIns4*P* at the wound site.

To specifically deplete PtdIns4P, avoiding potential side effects of drugs, we applied an FRB::FKBP rapamycin inducible dimerization system to deplete endogenous PtdIns4*P* (Hammond et al., 2012; Varnai et al., 2006; Fig. 2 G). This system involves fusing the FRB domain with the *S. cerevisiae* SAC1 phosphatase, which is capable of dephosphorylating PtdIns4*P* (Hammond et al., 2012). Before treatment with rapamycin, FRB::SAC1 was located in the cytosol, while tagBFP::P4M fusion with FKBP retained its normal localization (Fig. S2 A). After adding rapamycin, the fluorescence signal of tagBFP::P4M was significantly reduced (Fig. 2, H and I), possibly due to the degradation by SAC1. The PH::mKate2 also did not increase at the wounded membrane in animals expressing FRB::SAC1 after treatment with rapamycin compared to the control group (Fig. 2, H and I; and Fig. S2, A and B). Thus, this result supports the notion that PtdIns(4,5)*P*_2_ generation depends on the enrichment of PtdIns4*P* at the wound site.

The biochemical conversion of PtdIns4*P* to PtdIns(4,5)*P*2 on the plasma membrane requires the specific enzyme PtdIns4*P* 5-kinase (PI4P5K; Bulley et al., 2015; Fig. 2 A). Therefore, to examine the PI4P5K effect on the PtdIns(4,5)*P*_2_ generation, we knocked down *ppk-1*, which encodes PI4P5K in *C. elegans* (Weinkove et al., 2008). RNAi knockdown of *ppk-1* did not appear to affect mKate2::P4M accumulation, but it significantly reduced the accumulation of PH::GFP at the wound sites (Fig. S2, C–E), suggesting that PPK-1 is required for PtdIns(4,5)*P*_2_ generation but not the accumulation of PtdIns4*P*. Unfortunately, *ppk-1* loss of function in worms results in embryonic lethality (Weinkove et al., 2008), ruling out the possibility of generating null allele mutants for wounding experiments. To overcome this challenge, we created a mutant worm strain carrying *ppk-1(syb6134)*, in which two leucines (L) at the 218 and 226 positions were mutated into isoleucine (I; Fig. S2, F and G). This change was based on a previous study that showed the mutation of two leucines in human PI4P5K to isoleucine (occurring at positions 202 and 210) could reduce kinase activity (Shulga et al., 2012). As expected, the *ppk-1(syb6134)* mutant resulted in a reduced accumulation of PH::GFP at the wound sites (Fig. 2, J and K), suggesting that PPK-1 is required for PtdIns(4,5)*P*_2_ generation from PtdIns4*P* at the wound site.

The transcriptional reporter assay showed that *ppk-1* was expressed in multiple tissues, including the epidermis (Fig. S2 H), consistent with the previous report (Weinkove et al., 2008). We then determined whether PPK-1 contributes to PtdIns(4,5)*P*_2_ generation locally at the wound site by creating an epidermal expressed GFP::PPK-1 transgenic animal and imaging the signals before and after wounding. Before wounding, the GFP::PPK-1 formed small puncta and colocalized with the membrane marker myr::mKate2 (Fig. S2 I). After wounding, both GFP::PPK-1 and myr::mKate2 were specifically accumulated and colocalized at the wound site (Fig. S2 I). Time-lapse images showed that GFP::PPK-1 was explicitly recruited to the wounded membrane as early as 5 min after wounding (Fig. S2 J and Video 8). Importantly, the accumulated GFP::PPK-1 at the wound site could almost entirely colocalize with both mKate2::P4M and PH::mKate2 (Fig. 2, L and M; and Video 8). Further analysis of PPK-1 truncations (N, PIPK, ΔC, C) revealed that the PIPK domain is required and sufficient for its recruitment to the wound site (Fig. S2 K). Together, these results suggest that PPK-1 responds to membrane damage, recruits to the wound site through its PIPK domain, and promotes the local generation of PtdIns(4,5)*P*_2_ in membrane repair.

**Video 8.** **Representative single plane confocal time-lapse images showing recruitment of GFP::PPK-1 and mKate2::P4M to the wound site after needle wounding.** Time-lapse was taken for 60 min at 60 s interval. Frame rate: 7 frames per second. Pcol-19-mKate2::P4M(zjuSi333); Pcol-19-GFP::PPK-1(zjuSi367) transgenic animals were used for wounding and imaging. Scale bar: 10 µm. Related to Fig. 2.

### Wounding triggers the enrichment of the Golgi membrane to the wound site

**Figure S3.**
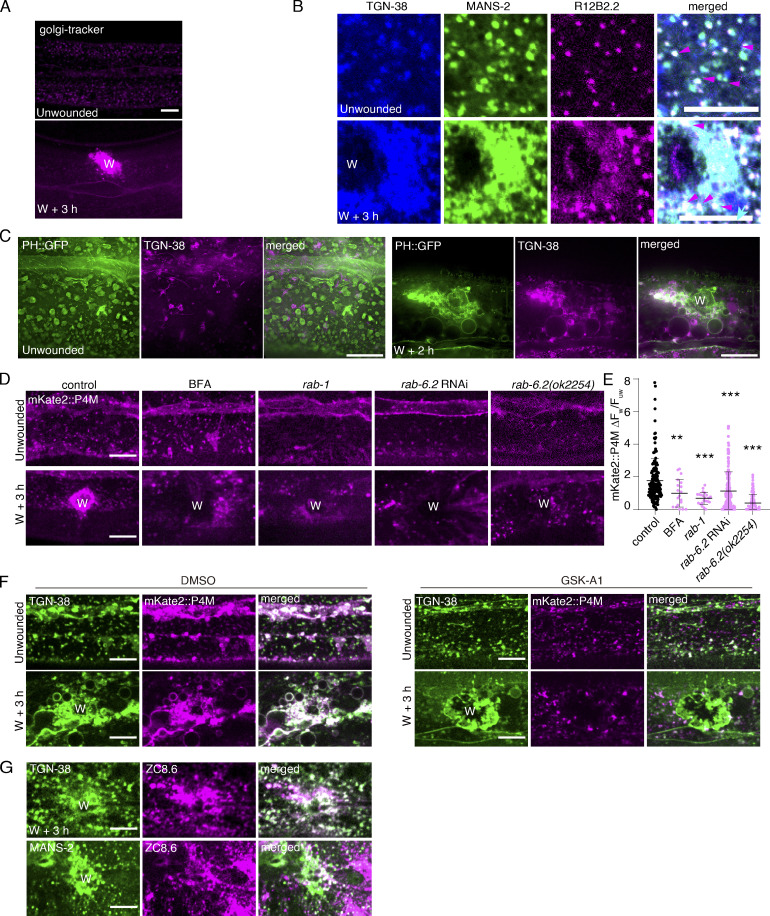
**Wounding triggers the enrichment of the Golgi membrane at the wound site, related to** Fig. 3**. (A)** Representative single-plane confocal images showing the localization of the Golgi apparatus (stained with Golgi-tracker-red) before and after wounding in N2 worms. “W” indicates the wound site. Scale bar: 10 µm. **(B)** Representative single-plane confocal images showing the colocalization of TGN-38::tagBFP, GFP::MANS-2, and mKate2::R12B2.2 before and after wounding. *Pcol-19-*TGN-38::tagBFP(*zjuSi280)*; P*col-19-*GFP::MANS-2(*zjuSi302*); *Pcol-19-*mKate2::R12B2.2(*zjuEx2075*) transgenic animals were used for wounding and imaging. Magenta arrows indicate the colocalized puncta. Scale bar: 10 µm. **(C)** Representative HiS-SiM single-plane images showing the colocalization of PH::GFP and TGN-38::tagBFP before and after wounding. P*col-19*-PH::GFP(*zjuSi175*); *Pcol-19-*TGN-38::tagBFP(*zjuSi280)* transgenic animals were used for wounding and imaging. “W” indicates the wound site. Scale bar: 10 µm. **(D)** Representative confocal images of mKate2::P4M with the treatment of *rab-1* RNAi*, rab-6.2* RNAi, and BFA (150 µg/ml), as well as in *rab-6.2(ok2254)* mutant animals before and after wounding. *Pcol-19-mKate2::P4M(zjuSi333)* transgenic animals were used for drug treatment, *Pcol-19-mKate2::P4M(zjuSi333);* P*col-19*-RDE-1(*juIs346); rde-1(ne219)* for RNAi treatments. Scale bar: 10 µm. **(E)** Quantitation analysis of the intensity ratio (ΔF_w_/F_uw_) for the different treatments shown in (E). The error bars represent the mean value ± SD (*n* = 156, 19, 22, 138, 134 from the left to the right), Mann–Whitney test, ***P < 0.001. **P < 0.01. **(F)** Representative confocal images showing TGN-38::tagBFP and mKate2::P4M with the treatment of GSK-A1 (100 μM) before and after wounding. *Pcol-19-*TGN-38::tagBFP(*zjuSi280); Pcol-19-mKate2::P4M(zjuSi333)* transgenic animals were used for wounding and imaging. Scale bar: 10 µm. **(G)** Representative single-plane confocal images showing the colocalization of mKate2::ZC8.6 with TGN-38::tagBFP or GFP::MANS-2. P*col-19-*TGN-38::tagBFP(*zjuSi280);* P*col-19-*mKate2::ZC8.6(*zjuEx2330),* P*col-19-*GFP::MANS-2(*zjuSi302*); P*col-19-*mKate2::ZC8.6(*zjuEx2320)* transgenic animals were used for wounding and imaging. Scale bar: 10 µm.

**Figure 3.**
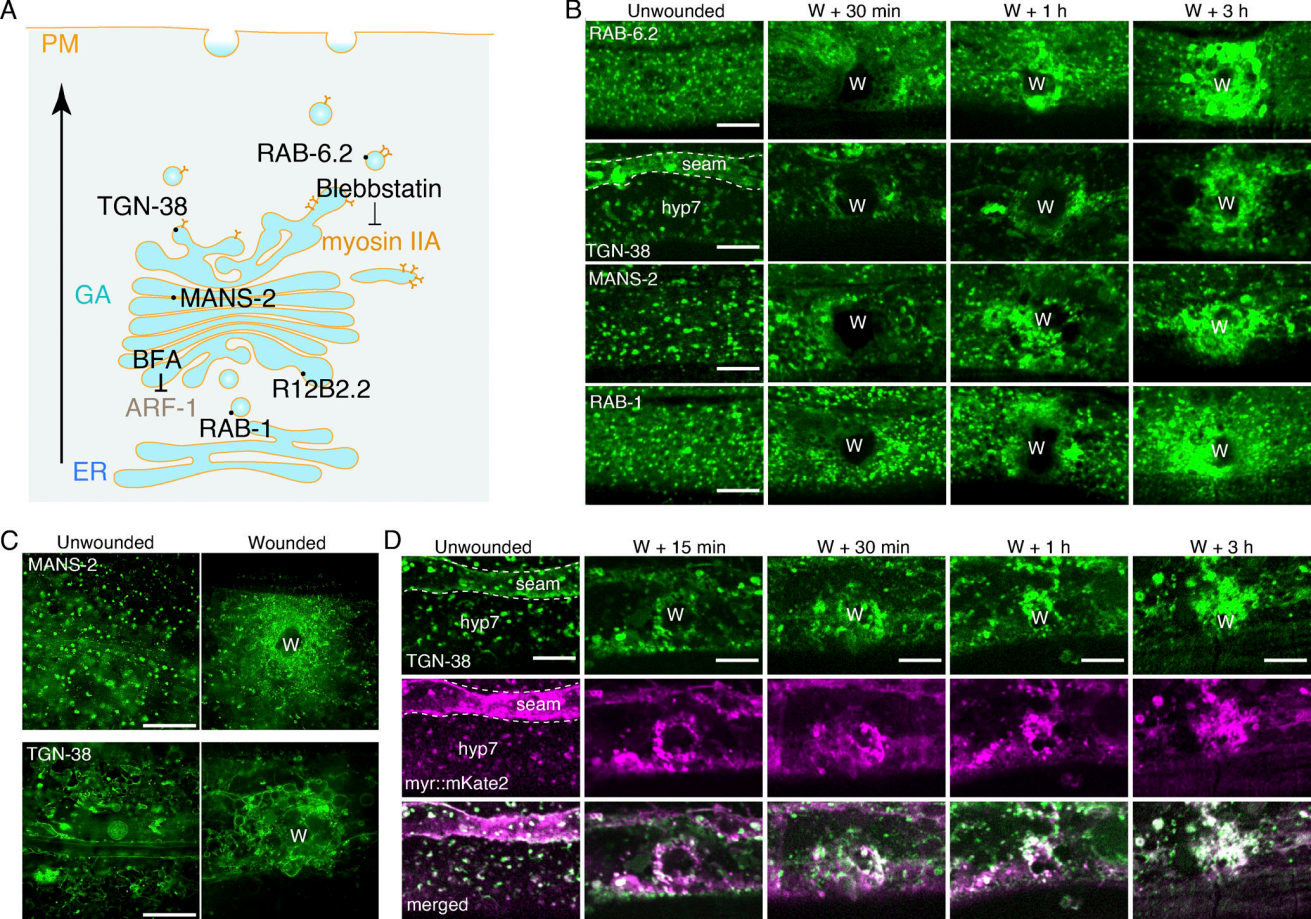
**Wounding triggers the accumulation of the Golgi membrane at the wound site. (A)** Diagram showing the location of Golgi apparatus compartment markers and the target of pharmacological inhibitors. **(B)** Representative confocal images showing the distribution of Golgi apparatus markers before and after wounding. The markers include GFP::RAB-1 (ERGIC), GFP::MANS-2 (medial), GFP::RAB-6.2 (trans), and TGN-38::tagBFP (TGN), respectively. P*col-19*-GFP::RAB-6.2(*zjuSi313*), P*col-19*-TGN-38::tagBFP(*zjuSi280*), P*col-19*-GFP::MANS-2(*zjuSi302*), and *Pcol-19-*GFP::RAB-1*(zjuSi253)* transgenic animals were used for wounding and imaging. “W” indicates the wound site. Scale bar: 10 µm. **(C)** HiS-SiM single-plane images showing the accumulation of TGN-38::tagBFP and GFP::MANS-2 at the wound site. P*col-19*-TGN-38::tagBFP(*zjuSi280*) and P*col-19*-GFP::MANS-2(*zjuSi302*) transgenic animals were used for wounding and imaging. Scale bar: 10 µm. **(D)** Representative single-plane confocal images showing the colocalization of TGN-38::tagBFP and myr::mKate2 before and after wounding. *Pcol-19-*TGN-38::tagBFP(*zjuSi280)*; P*col-19-*myr::mKate2(*zjuSi46)* transgenic animals were used for wounding and imaging. “W” indicates the wound site. Scale bar: 10 µm. See also Fig. S3 and Video 9.

PtdIns4P are largely localized on the Golgi apparatus, which is a key organelle involved in protein modification, lipidation, and intracellular membrane trafficking (Glick and Nakano, 2009); however, its role in responding to wounding, providing PtdIns4P, and facilitating membrane repair is unclear. To explore this, we stained the animals with the Golgi tracker, a specific ceramide dye that stains the Golgi apparatus (Meisslitzer-Ruppitsch et al., 2011; Pagano, 1989). The Golgi tracker signal displayed punctated in the entire hyp7 cell before wounding and appeared to accumulate at the wound sites after wounding (Fig. S3 A). To validate this observation, we generated several fluorescent fusion reporters to label the different membrane structures of the Golgi apparatus, such as RAB-1 (ERGIC), MANS-2 (medial Golgi), TGN-38 (trans-Golgi network), and RAB-6.2 (trans-Golgi; Lippincott-Schwartz et al., 2000; Fig. 3 A). Unlike the typical Golgi apparatus stacks located around the perinuclear region (Bui et al., 2021), these Golgi apparatus markers were punctated and distributed in the cytosol of the entire hyp7 cell (Fig. 3 B). After wounding, these Golgi membrane markers were specifically enriched at the wound sites (Fig. 3 B and Video 9). Triple fluorescence of the Golgi apparatus markers using TGN-38::tagBFP, GFP::MANS-2, and mKate2::R12B2.2 (ortholog of human BLZF1, a cis-Golgi marker; Fig. 3 A) showed that all of them were colocalized in the epidermal cell and enriched at the wound sites (Fig. S3 B). These results suggest that wounding triggers the Golgi membrane enrichment at the wound site.

**Video 9.** **Representative single plane confocal time-lapse images showing recruitment of GFP::MANS-2 to the wound site after needle wounding.** Time-lapse was taken for 80 min at 30 s interval. Frame rate: 20 frames per second. Pcol-19-GFP::MANS-2(zjuSi302) transgenic animals were used for wounding and imaging. Scale bar: 10 µm. Related to Fig. 3.

We further examined the Golgi membrane enrichment at the wound site by performing HiS-SIM imaging of GFP::MANS-2 and TGN-38::tagBFP before and after wounding. GFP::MANS-2 formed fragmented punctate before wounding and accumulated at the wound sites (Fig. 3 C). TGN-38::tagBFP located at the intracellular membrane compartment before wounding and formed larger cysts with interconnected vesicle structures at the wound site (Fig. 3 C), indicating the Golgi apparatus-derived membrane structure formed a membrane patch to repair the wounded membrane. Moreover, TGN-38::tagBFP showed strong colocalization with membrane marker myr::mKate2 at the wound site at different time points after wounding (Fig. 3 D). Together, these results suggest that membrane wounding triggers the enrichment of the Golgi membrane at the wound site.

### The enrichment of the Golgi membrane at the wound site is required for membrane repair

**Figure 4.**
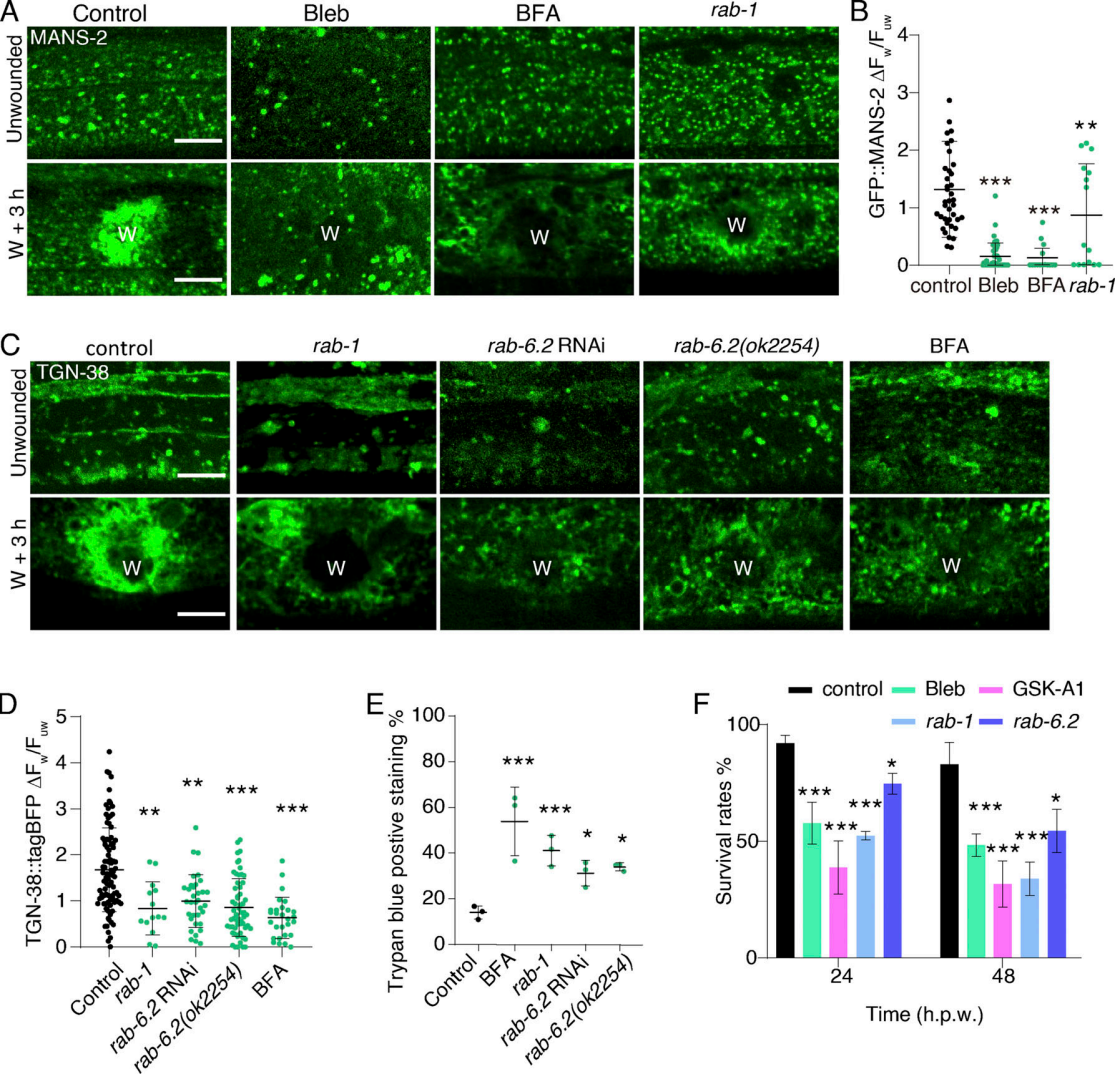
**The accumulation of the Golgi membrane at the wound site is required for membrane repair. (A)** Representative confocal images showing TGN-38::tagBFP before and after wounding with the treatment of *rab-1* and *rab-6.2* RNAi, as well as *rab-6.2(ok2254)* deletion mutant. P*col-19-*TGN-38::tagBFP(*zjuSi280*) for RNAi, and P*col-19-*TGN-38::tagBFP(*zjuSi280*); P*col-19*-RDE-1(*juIs346); rde-1(ne219)* transgenic animals were used for wounding and imaging. Scale bar: 10 µm. **(B)** Quantitation analysis of the intensity ratio (ΔF_w_/F_uw_) of TGN-38::tagBFP after wounding (A). Error bars represent the mean value ± SD (*n* = 93, 15, 34, 58, 28 from the left to the right). One-way ANOVA multiple comparisons, ***P < 0.001. **P < 0.01. **(C)** Representative confocal images showing GFP::MANS-2 before and after wounding with the treatment of Blebbistatin (20 µM) and BFA (150 µg/ml). P*col-19*-GFP::MANS-2(*zjuSi302*) for drug treatment were used for wounding and imaging. Scale bar: 10 µm. **(D)** Quantitation analysis of the intensity ratio (ΔF_w_/F_uw_) of GFP::MANS-2 after wounding (C). Error bars represent the mean value ± SD (*n* = 33, 45, 15, 15 from the left to the right). One-way ANOVA multiple comparisons, ***P < 0.001. **(E)** Quantitation analysis of the percentage of trypan blue staining of worms at 6 h after wounding treated with BFA (150 µg/ml), *rab-1* RNAi, and *rab-6.2* RNAi, as well as *rab-6.2(ok2254)* deletion mutant. N2 strain was used for drug treatments. P*col-19*-RDE-1(*juIs346*); *rde-1(ne219)* strain was used for RNAi treatment. The error bars represent the mean value. ± SD (*n* = 3), Student’s *t* test, *P < 0.05. ***P < 0.001. **(F)** Quantitation analysis of the survival rates (%) of animals treated with Blebbistatin (20 µM), *rab-1* RNAi, *rab-6.2* RNAi, and GSK-A1(100 μM) after wounding. N2 for drug treatments and P*col-19*-RDE-1(*juIs346); rde-1(ne219)* transgenic animals for RNAi treatments, wounding and survival rate quantification. The error bars represent the mean value ± SD (*n* = 3), Student’s *t* test, ***P < 0.001. *P < 0.05. See also Fig. S3.

We then investigated how the Golgi apparatus accumulated to the wounded membrane by examining the conventional route (delivery in vesicular tubular carriers to the plasma membrane) of the Golgi apparatus transport pathway (Stalder and Gershlick, 2020). We used Blebbistatin to inhibit myosin IIA, which is required for the transport of the Golgi membrane (Fig. 3 A). Worms treated with Blebbistatin showed a more punctated GFP::MANS-2 signal and reduced accumulation at the wound site (Fig. 4, A and B). Additionally, animals were treated with a protein transport inhibitor Brefeldin A (BFA), which induces retrieval of the Golgi apparatus to the ER by interfering with anterograde transportation (Bershadsky and Futerman, 1994; Lippincott-Schwartz et al., 1991; Fig. 3 A), also inhibited the GFP::MANS-2 and TGN-38::tagBFP accumulation to the wound site (Fig. 4, A–D). Together, these observations suggest that the enrichment of the Golgi membrane at the wound sites depends on the anterograde transport pathway.

We then determined whether the accumulation of the Golgi membrane contributes to the membrane repair. We performed staining with TryB and found that BFA treatment significantly increased the ratio of TryB staining (Fig. 4 E). RAB-1 is well-known to function in the anterograde transport from the ER to the Golgi apparatus (Barr, 2009; Kiral et al., 2018). RNAi knockdown of *rab-1* resulted in no TGN-38::tagBFP and GFP::MANS-2 accumulation at the wound site (Fig. 4, C and D). Epidermal-specific RNAi knockdown of *rab-1* increased the ratio of TryB-positive staining (Fig. 4 E). We further found that in *rab-6.2* RNAi knockdown or utilized its loss-of-function of *rab-6.2(ok2254)* animals*,* the accumulation of the TGN-38::tagBFP was inhibited at the wound site (Fig. 4, C and D). Consistently, the ratio of TryB-positive staining was increased in these mutant animals (Fig. 4 E). These results suggest that the enrichment of the Golgi membrane to the wound site is required for membrane repair. The hyp7 contains multinuclear and covers the entire epidermal anatomy, and defects of membrane repair result in a significant consequence for organism survival (Chisholm and Xu, 2012; Xu and Chisholm, 2011). We observed that treatment with Blebbistatin, GSK-A1, or knockdown of *rab-1* and *rab-6.2*, all led to a significant decrease in post-wounding survival ratio 24 and 48 h after wounding (Fig. 4 F). Together, these results support that the Golgi membrane enrichment at the wound site contributes to membrane repair and animal survival after wounding.

### The Golgi membrane enrichment provides PtdIns4*P* for PtdIns(4,5)*P*_2_ local generation in membrane repair

**Figure 5.**
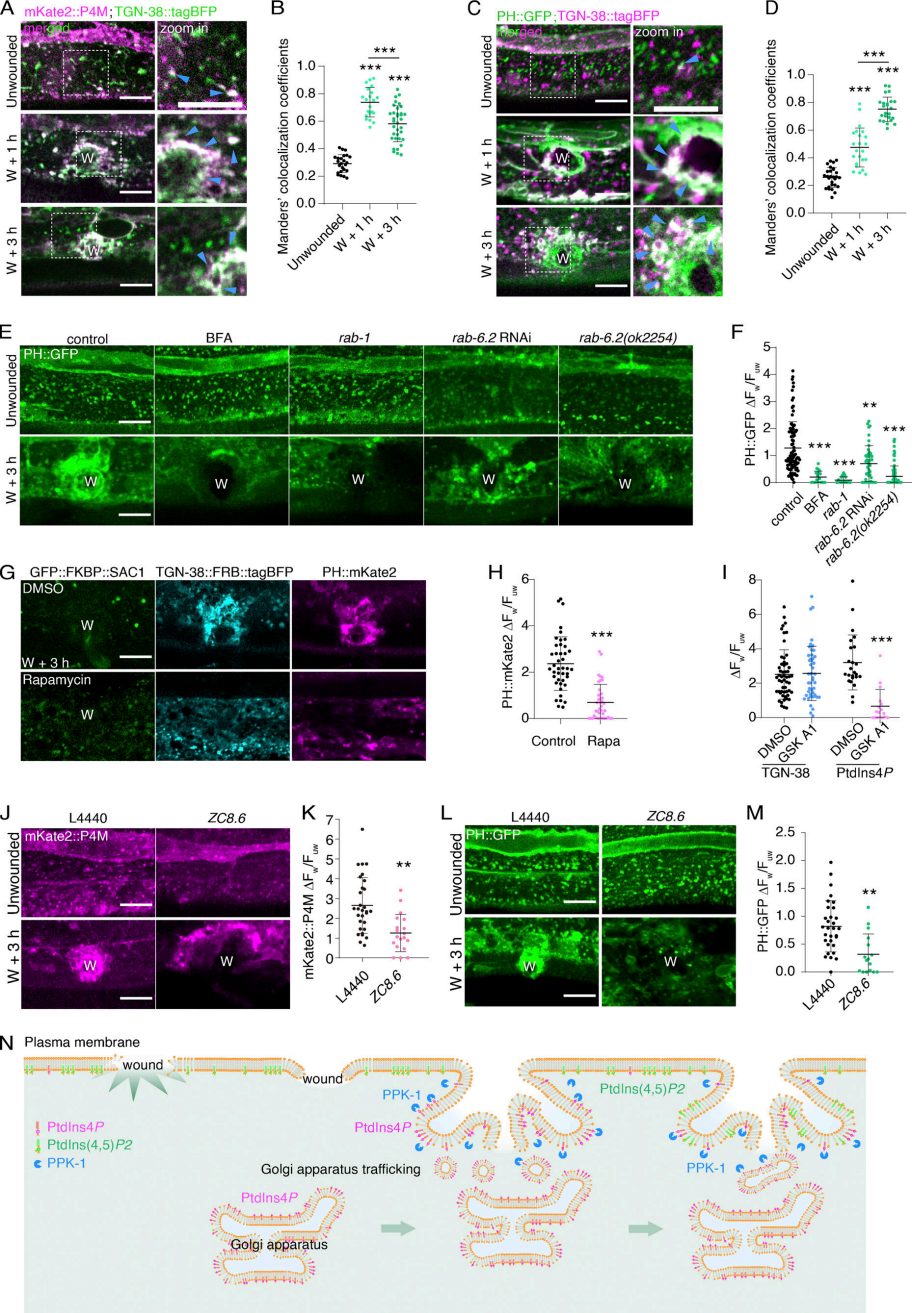
**The Golgi membrane enrichment provides PtdIns4*P* for PtdIns(4,5)*P***_**2**_ **local generation in membrane repair. (A)** Representative single-plane confocal images of mKate2::P4M and TGN-38::tagBFP before and after wounding. P*col-19*-mKate2::P4M(*zjuSi333*); P*col-19*-TGN-38::tagBFP(*zjuSi280*) transgenic animals were used for wounding and imaging. “W” indicates the wound site. The white dotted box indicates the zoom-in region. Magenta arrows point to the colocalized puncta. Scale bar: 10 µm. **(B)** Mander's colocalization analysis of mKate2::P4M and TGN-38::tagBFP before and after wounding. The error bars represent the mean value ± SD (*n* = 24, 22, 60 from the left to the right), Mann*—*Whitney test, ***P < 0.001. **(C)** Representative single-plane confocal images showing the recruitment of PH::GFP and TGN-38::tagBFP before and after wounding. P*col-19-*PH::GFP(*zjuSi175)*; P*col-19*-TGN-38::tagBFP(*zjuSi280*) transgenic animals were used for wounding and imaging. The white dotted box indicates the zoom-in region. Magenta arrows indicate the colocalized puncta. Scale bar: 10 µm. **(D)** Mander’s colocalization analysis of PH::GFP and TGN-38::tagBFP before and after wounding. The error bars represent the mean value ± SD (*n* = 28, 23, 24 from the left to the right), Mann*—*Whitney test, ***P < 0.001. **(E)** Representative confocal images showing PH::GFP before and after wounding with the treatments of BFA (150 µg/ml), *rab-1* RNAi, and *rab-6.2* RNAi, as well as *rab-6.2(ok2254)* mutant. P*col-19*-PH::GFP(*zjuSi175*) for drug treatments and P*col-19*-PH::GFP(*zjuSi175*);P*col-19*-RDE-1(*juIs346*); *rde-1(ne219)* for RNAi treatment. Scale bar: 10 µm. **(F)** Quantitation analysis of the intensity ratio (ΔF_w_/F_uw_) of PH::GFP in BFA (150 µg/ml) and *rab-1*, *rab-6.2* RNAi, and *rab-6.2(ok2254)* mutant animals after wounding (E). The error bars represent the mean value ± SD (*n* = 91, 19, 24, 44, 93 from the left to the right). One-way ANOVA multiple comparisons, ***P < 0.001. **P < 0.01. **(G)** Representative confocal images of TGN-38:: FRB::tagBFP and PH::mKate2 accumulation at 3 h after wounding after rapamycin (100 µM) treatment. *Pcol-19-PH::mKate2(zjuSi321)* II; *Pcol-19-TGN-38::FRB::tagBFP*; *Pcol-19-GFP::FKBP::SAC1(zjuEx2307)* animals were used for wounding and imaging. Scale bar: 10 µm. **(H)** Quantitation analysis of the intensity ratio (ΔF_w_/F_uw_) of PH::mKate2 in rapamycin and DMSO-treated worms (G). The error bars represent the mean value ± SD (*n* = 13 and 16). Mann–Whitney test, ***P < 0.001. **(I)** Quantitation analysis of the intensity ratio (ΔF_w_/F_uw_) of TGN-38::tagBFP and mKate2::P4M with GSK-A1 (100 μM) treatment. The error bar represents the mean value ± SD (*n* = 54, 47, 24, 17 from the left to the right). Mann*—*Whitney test, ***P < 0.001. **(J)** Representative confocal images of mKate2::P4M before and after wounding with the treatment of *zc8.6* RNAi. P*col-19*-mKate2::P4M(*zjuSi333*); P*col-19*-RDE-1(*juIs346*); *rde-1(ne219)* animal was used for wounding and imaging. Scale bar: 10 µm. **(K)** Quantitation analysis of the intensity ratio (ΔF_w_/F_uw_) of mKate2::P4M. The error bar represents the mean value ± SD (*n* = 30 and 19), Mann–Whitney test, **P < 0.01. **(L)** Representative confocal images of PH::GFP before and after wounding with the treatment of *zc8.6* RNAi. P*col-19*-PH::GFP(*zjuSi175*); P*col-19*-RDE-1(*juIs346*); *rde-1(ne219)* animal was used for wounding and imaging. Scale bar: 10 µm. **(M)** Quantitation analysis of the intensity ratio (ΔF_w_/F_uw_) of PH::GFP. The error bar represents the mean value ± SD (*n* = 31 and 15), Mann–Whitney test, **P < 0.01. **(N)** Illustration of the proposed model on how the Golgi apparatus-originated translocation to the wound site to deliver PtdIns4*P* for PtdIns(4,5)*P*_2_ generation in *C. elegans* epidermal membrane repair. See also Fig. S3 and Video 10.

Notably, the wounding-triggered accumulation of the mKate2::P4M was colocalized with TGN-38::tagBFP, suggesting PtdIns4*P* was indeed localized to the Golgi membrane (Fig. 5, A and B). Before wounding, TGN-38::tagBFP and PH::GFP were not colocalized, but after wounding, they were strongly colocalized within a large vesicle membrane-like structure at the wound site (Fig. 5, C and D; and Fig. S3 C and Video 10). We thus investigated whether the Golgi membrane accumulation contributed to the PtdIns(4,5)*P2* generation at the wounded membrane. We found that BFA treatment significantly reduced the accumulation of mKate2::P4M at the wound site (Fig. S3, D and E). Moreover, no PH::GFP intensity was increased at the wound sites after BFA treatment (Fig. 5, E and F). Similarly, RNAi knockdown of *rab-1* and *rab-6.2, rab-6.2* mutant, all reduced the accumulation of mKate2::P4M and PH::GFP at the wound site (Fig. 5, E and F; and Fig. S3, D and E). These results suggest that the enrichment of the Golgi membrane is necessary for the generation of PtdIns(4,5)*P*_2_ at the wound site. We then determined whether PtdIns4*P* on the Golgi membrane is required for PtdIns(4,5)*P*_2_ generation by expressing TGN-38::FRB and FKBP::SAC-1 in the epidermal cell to specifically deplete PtdIns4*P* on the Golgi membrane (Dickson et al., 2014). We observed that the accumulation of PH::mKate2 was significantly reduced after adding the rapamycin (Fig. 5, G and H), suggesting PtdIns4*P* on the Golgi membrane is necessary for PtdIns(4,5)*P*_2_ generation at the wounded membrane.

**Video 10.** **Representative single plane confocal time-lapse images showing recruitment of PH::GFP and TGN-38::tagBFP (in red) after needle wounding.** Time-lapse was taken for 70 min at 30 s interval. Frame rate: 52 frames per second. Pcol-19-PH::GFP(zjuSi175); Pcol-19-TGN-38::tagBFP(zjuSi280) transgenic animals were used for wounding and imaging. Scale bar: 10 µm. Related to Fig. 5.

Although GSK-A1 treatment did not affect TGN-38::tagBFP accumulation at the wound site, it significantly inhibited the accumulation of mKate2::P4M (Fig. 5 I and Fig. S3 F), which is consistent with the decrease in PtdIns(4,5)*P*_2_ generation after GSK-A1 treatment (Fig. 2, D and E). GSK-A1 specifically inhibits the activity of PI4Kα, implying that the Golgi apparatus localized PI4K may be involved in this process. We identified a *C. elegans* PI4Kα orthology ZC8.6, whose mKate2 fusion protein is colocalized with TGN-38::tagBFP and MANS-2::GFP (Fig. S3 G), suggesting ZC8.6 is a Golgi apparatus-specific PI4Kα. Importantly, RNAi knockdown of *ZC8.6* decreased the mKate2::P4M and the PH::GFP signal at the wound site (Fig. 5, J–M). Together, these results suggest that the Golgi apparatus-specific PI4Kα ZC8.6 contributes to PtdIns4*P* synthesis and PtdIns(4,5)*P*_2_ generation during membrane repair.

The present study demonstrated that the Golgi apparatus responds to wounding to facilitate membrane repair by providing a membrane source, in which PtdIns4*P* is used as a substrate for the generation of PtdIns(4,5)*P*_2_ at the wounded site (Fig. 5 N). Beyond advancing our in vivo understanding of the materials used by cells for post-wounding membrane repair, our study highlights a function of the Golgi apparatus in acute response to cell stress. The accumulation of the Golgi membrane at the wound site is part of a complex process that may also involve the delivery of other lipids and enzymes required for membrane repair. It is worth noting that the Golgi apparatus in the hyp7 cell of *C. elegans* are disorganized and fragmented, which is similar to that observed in cancers, as opposed to the typical stack structure in eukaryotic cells. This may highlight the importance of the Golgi structure in cancer cell survival mechanisms upon immune attack by the T cells. Nevertheless, our study broadens the known functional capacity of the Golgi apparatus and warrants further investigations into its role in membrane repair processes and its potential involvement in other cellular responses to stress in a physiological context.

## Materials and methods

### *C. elegans* strains

All *C. elegans* were grown in NGM plates at a temperature of 20–25°C. For injection, N2 worms were grown to the young adult stage and placed onto 4% agarose pads inside halogen oil to minimize mobility. Worms were placed under an injection microscope (Nikon Eclipse Ti) and injected with a plasmid mix composed of 10 ng/μl gene of interest codon plasmid, 50 ng/μl co-injection marker, 50 ng/μl empty vector (blueprint plasmid) for an extrachromosomal array, 50 ng/μl SCI (single-copy-insertion) template plasmid, 50 ng/μl Cas9 with sgRNA coding plasmid, and with 2.5–5 ng/μl co-injection markers for SCI strains (Xu et al., 2016). New strains were constructed using standard procedures and were genotyped by PCR or sequencing. All strains and plasmids are listed in Table S2 and S3, respectively.

### Cloning

*C. elegans* genomic DNA and cDNA were used as the template for cloning. The cloned genes were transformed into DH5α vectors and were seeded to bacterium plates with respective antibiotics at 37ºC overnight. Non-*C. elegans* coding sequences were generated by Tsingke Biotechnology Co., Ltd. and the codons were optimized using EMBOSS Backtranseq tool (ebi.ac.uk) to ensure efficient translation.

### Live imaging

The live imaging experiments were performed using young adult stage worms that were synchronized and grown. Prior to imaging, worms were picked and immobilized on 4% agarose pads using a small drop of 12 mM levamisole. Worms are mounted to Nikon Eclipse Ni and Z-stack images were acquired with a 100× magnification using a Nikon Plan Apo 100 X/1.40 oil microscope, with a 0.5 μm interval between each image.

### HIS-SIM imaging

The HIS-SIM was performed according to the method previously described (Huang et al., 2018). The commercial HIS-SIM used in this study was updated by Guangzhou Computational Super-resolution Biotech Co., Ltd. The images were acquired and processed using this system. For additional resolution improvement, the images were reconstructed by sparse deconvolution (Zhao et al., 2022). Briefly, worms were picked and immobilized on 4% agarose pads using a small drop of 12 mM levamisole. Images were acquired with a 100X /1.5 NA immersion objectives (Olympus). Image acquisition was carried out using IMAGER software. GFP were excited with 488 laser and a 525/20 nm filter. BFP was excited with 405 nm laser and a 450/50 nm filter.

### Adult epidermal-specific RNAi

Adult epidermal-specific RNAi was performed as previously described (Xu and Chisholm, 2011). Briefly, synchronized L1 stage worms carrying (*rde-1(ne219); Pcol-19-RDE-1(juls346)*) were placed onto HT115 *Escherichia coli* seeded NGM containing 1 mM IPTG and Carbenicillin. The worms were then incubated at 20°C until they reached the young adult stage to be subjected to further experiments.

### Drug treatment

Drugs were diluted in DMSO to make 10-mM master solution. The working solution was produced by diluting the master solution with M9 buffer seeded with OP50 to reach the working concentration, as indicated in each experiment. 100 μl aliquot of the working solution was placed on 96-well plates. Young adult worms that had been wounded were picked and placed into the drug solution and incubated at 20°C until the required time points for imaging. The DMSO group was taken as the control group.

### Wounding

Microinjection needles were pulled from the capillary tube using a Narishige PC-10 Micropipette puller. For needle wounding, young adult stage worms were synchronized and placed under a dissection microscope. Two stab wounds were punctured using micro-injection needles for each worm as described previously (Wijaya et al., 2020). For laser wounding, worms were placed on 4% agar pads and were immobilized using 12 mM levamisole before being mounted on a confocal microscope and wounded using a Micropoint UV laser integrated with the spinning disk confocal microscope.

### Trypan blue staining

Wounded young adult stage worms were incubated until the respective time points for observation time points. Afterward, the worms were immersed in a 0.6% (weight/volume) solution of trypan blue (T6146; Sigma-Aldrich) for 60 min. Observations were carried out under an upright microscope (Nikon Eclipse Ni). Number of TryB stained worms/total number of wounded worms × 100% were calculated for each experimental repeats to obtain the percentage of TryB positive staining percentage at a time point after wounding.

### Golgi tracker-red staining

The synchronized young adult stage worms were picked into 96-well plates containing a solution of the Golgi tracker-red (C1043; Beyotime) that was diluted 1:100 in BSA. The staining process was carried out according to the manufacturer’s instructions by submerging the worm in the staining solution for 20 min. The worms were then washed 3 × with M9 solution and subjected to wounding and imaging under the confocal microscope.

### Image analysis

The fluorescence signals were quantified using ImageJ software (http://rsb.info.nih.gov/ij/). For each worm, boundaries were drawn around the recruited fluorescence signal to quantify the absolute intensity (I_w_). Two regions of the unwounded/wounded region were selected, and their average intensities were calculated to measure the intensity of the unwounded/wounded region (I_uw_/I_w_). The background intensity of each image was also quantified (I_bg_). For each wound, Iw−Iuw/Iuw−Ibg was calculated to obtain the recruitment fluorescence signal (ΔF_w_/F_uw_). Intensities measured were analyzed using GraphPad Prism 9 to generate a line graph.

### Colocalization analysis

The colocalization analysis was conducted by calculating Manders’ colocalization coefficient (MCC) as described (Schneider et al., 2012). Briefly, the MCC value ranges from −1 to 1, where a value of 1 indicates complete colocalization, a value of 0 indicates no colocalization, and a value of −1 indicates negative colocalization. For analysis, the regions of interest (ROIs, 13.8 × 13.8 μm) were selected in the captured images. The region around the wound site was selected for wounded worms as the ROI. The quantitative value of MCC was calculated in ImageJ by the following options: Analyze-Colocalization-Coloc 2.

### FRAP analysis

For each image, the punctate signals were bleached using 100% laser transmission with an argon laser power is 30 mW, with 50% (15 mW) 405 nm laser. The fluorescence intensity was then imaged at 10-s intervals for 20 min and analyzed using ImageJ software. The relative fluorescence intensity of the photobleached region at each time point (F_t_), the region before photobleaching (F_u_), and the region of background (F_b_) were measured. The $\left(F_{t}-F_{b}\right)/\left(F_{u}-F_{b}\right)$ values were plotted against time to obtain the fluorescence recovery after the photobleaching (FRAP) curve. The mobile fraction (F_m_) was calculated to evaluate the FRAP efficiency by the equation: $Fm=\left(F_{\max}-F_{b}\right)/\left(F_{u}-F_{b}\right)\approx \left(F_{20}-F_{b}\right)/\left(F_{u}-F_{b}\right)$, where F_20_ is the relative fluorescence intensity 20 min after photobleaching.

### Quantitative analysis

All experiments were conducted a minimum of three times to ensure accuracy and reproducibility. Statistical analyses were performed using GraphPad Prism 9. The average of the individual data was represented on the Y-axis of the graph, with the SD represented as an error bar. For two-way comparisons, Student’s *t* test (two-tailed unpaired) or Mann–Whitney test were used. For multiple comparisons, a one-way ANOVA test was used. Data distribution was assumed to be normal but was not formally tested. The results were considered statistically significant if P < 0.05. Statistically significant results were indicated with asterisks, where * represents P < 0.05, ** represents P < 0.01, and *** represents P < 0.001. At least 10 different wound sites were quantified in each experiment, and each experiment was repeated at least twice.

### Online supplemental material

Fig. S1 shows the PtdIns4P accumulation and PtdIns(4,5)P2 generation at the wound site. Fig. S2 shows that PtdIns(4,5)P2 generation depends on PtdIns4P, PI4K, and PI4P5K. Fig. S3 shows that wounding triggered the accumulation of the Golgi membrane, which provides PtdIns4P for PtdIns(4,5)P2 generation in membrane repair. Table S1 lists all the reagents used in this study. Table S2 lists the *C. elegans* strains used in this study. Table S3 lists the plasmids used in this study. Table S4 lists the primers used in this study. Video 1 shows representative PH::GFP signal in *C. elegans* epidermal cell before wounding. Video 2 shows recruitment of PH::GFP to the wound site after needle wounding. Video 3 shows recruitment of PH::GFP and myr::mKate2 to the wound site after needle wounding. Video 4 shows His-SIM time-lapse shows the recruitment of PH::GFP after needle wounding. Video 5 shows representative mKate2::P4M signal in unwounded *C. elegans* epidermal cell. Video 6 shows recruitment of PH::GFP and mKate2::P4M after needle wounding. Video 7 shows dynamic recruitment of PH::GFP and mKate2::P4M after needle wounding. Video 8 shows recruitment of GFP::PPK-1 and mKate2::P4M to the wound site after needle wounding. Video 9 shows recruitment of GFP::MANS-2 to the wound site after needle wounding. Video 10 shows recruitment of PH::GFP and TGN-38::tagBFP (in red) after needle wounding.

## Supplementary Material

Table S1 lists reagents used in this study.

Table S2 lists *C. elegans* strains used in this study.

Table S3 lists plasmids used in this study.

Table S4 lists primers used in this study.

## Data Availability

The data reported in this article are available in the published article and its online supplemental material or can be accessed from the Dryad dataset https://doi.org/10.5061/dryad.rbnzs7hgg. The plasmids and *C. elegans* strains are available from the corresponding author upon request.
