# Supplementary material for: Triggered Golgi membrane enrichment promotes PtdIns(4,5)P2 generation for plasma membrane repair
Source: J Cell Biol. 2023 May 9;222(8):e202303017. doi: 10.1083/jcb.202303017 (PMC10176212; doi:10.1083/jcb.202303017)
Supplement: Table S1 — lists reagents used in this study. [file JCB_202303017_TableS1.docx]

**Table S1.** List of reagents used in this study

| **Reagents** | **Source** | **Identifier** |
| --- | --- | --- |
| **Bacterial Strains** |  |  |
| OP50 | CGC | N/A |
| HT115 | Shanghai Yuanye | N/A |
| *Trelief*^®^5α Chemically Competent Cell | Tsingke | TSC-C01 |
| DB3.1 | WEIDI | DL1040 |
| **Chemicals** |  |  |
| Agarose | Tsingke | TSJ001 |
| M5 Gel-red | Mei5 | MF079-01 |
| Ethylenediamine tetraacetic acid (EDTA) | Sigma | E9884 |
| TRIS | Life Science | 0487C397 |
| Sodium chloride | Sigma | V900058 |
| Bacto Agar | BD | 214010 |
| Bacto Peptone | BD | 211677 |
| Cholesterol | Sigma | C8667 |
| Calcium chloride dihydrate | Sigma | C7902 |
| Magnesiumsulfate heptahydrate | Sigma | M1880 |
| Potassiumphosphate monobasic | Sigma | V900041 |
| Potassium phosphate dibasic | Sigma | V900050 |
| Sodium phosphate dibasic | Sigma | V900061 |
| Isopropyl beta-D-thiogalactoside | Sigma | V900061 |
| Carbenicillin Na_2_ | INALCO | 1758-9317 |
| Tryptone | OXOID | CM0129 |
| Yeast extract | OXOID | LP0021 |
| Potassium chloride | Sigma | V900068 |
| Golgi Tracker-red | Beyotime | C1043 |
| Trypan Blue | Sigma | T6146 |
| Phenyl Arsine Oxide (PAO) | Sigma | 637-03-6 |
| Brefeldin A (BFA) | Sigma | 2350-15-6 |
| GSK-A1 | MCE | HY-125118 |
| KDU-691 | MCE | HY-12912 |
| BQR-695 | MCE | HY-18748 |
| Blebbistatin | MCE | HY-13813 |
| **Critical Commercial Assays** |  |  |
| 2X Phanta Max Master Mix | Vazyme | P525-AA |
| I-5 2X High-Fidelity Master Mix | MCLAB | I5HM-200 |
| ClonExpress II One Step Cloning Kit | Vazyme | C112-02 |
| ClonExpress MultiS One Step Cloning Kit | Vazyme | C113-02 |
| Gateway LR clonase II | Invitrogen | 56484 |
| 10X KLD Enzyme Mix | NEB | M0554S |
