# Supplementary material for: Triggered Golgi membrane enrichment promotes PtdIns(4,5)P2 generation for plasma membrane repair
Source: J Cell Biol. 2023 May 9;222(8):e202303017. doi: 10.1083/jcb.202303017 (PMC10176212; doi:10.1083/jcb.202303017)
Supplement: Table S2 — lists C. elegans strains used in this study. [file JCB_202303017_TableS2.docx]

**Table S3.** List of plasmids used in this study

| **Plasmid DNA** | **Source** | **Identifier** |
| --- | --- | --- |
| pDEST5605-SEC-P*col-19*-GFP::­­­RAB-1 | This paper | pSX2259 |
| pCFJ201-SEC-P*col-19*-PH::GFP | This paper | pSX2349 |
| pCR8-P*col-19*-GBF-1::mKate2 | This paper | pSX2412 |
| pDEST340-SEC-P*col-19*-TGN-38::tagBFP | This paper | pSX2476 |
| pCFJ210-SEC-P*col-19*-GFP::RAB-6.2 | This paper | pSX2476 |
| pDEST5605-SEC-P*col-19*-GFP::MANS-2 | This paper | pSX2530 |
| pDEST5605-loxp-sec-loxp-P*col-19*-PH::mKate2 | This paper | pSX2588 |
| P*ppk-1-*GFP | This paper | pSX2639 |
| pDEST883-sec-p*col-19*-mKate2::P4M(SidM) | This paper | pSX2696 |
| pCR8-P*col-19*-GFP::FKBP1A(FKBP 2-108AA)::SAC1(2-517AA) | This paper | pSX2783 |
| pCR8-P*col-19*-P4M::FKBP12(mTOR)::linker::tagBFP | This paper | pSX2785 |
| pDEST340-SEC-P*col-19*-GFP::PPK-1 | This paper | pSX2918 |
| pCR8-P*col-19*-TGN-38::FKBP12(mTOR)::linker::tagBFP | This paper | pSX2943 |
| pCR8-P*col-19*-mKate2::R12B2.2 | This paper | pSX3020 |
| pCR8-P*col-19-*GFP::PPK-1(1-453) | This paper | pSX3135 |
| pCR8-P*col-19*-GFP::PPK-1(C) | This paper | pSX3136 |
| pCR8-P*col-19*-PPK-1(1-453)::GFP | This paper | pSX3152 |
| pCR8-P*col-19-*PPK-1(1-83)::GFP | This paper | pSX3217 |
| pCR8-P*col-19-*PPK-1(84-453)::GFP | This paper | pSX3218 |
| pCR8-P*col-19*-mKate2::ZC8.6 | This paper | pSX3266 |
