# Supplementary material for: Triggered Golgi membrane enrichment promotes PtdIns(4,5)P2 generation for plasma membrane repair
Source: J Cell Biol. 2023 May 9;222(8):e202303017. doi: 10.1083/jcb.202303017 (PMC10176212; doi:10.1083/jcb.202303017)
Supplement: Table S3 — lists plasmids used in this study. [file JCB_202303017_TableS3.docx]

**Table S2.** List of *C. elegans* strains used in this study

| **Genotype** | **Source** | **Strain** |
| --- | --- | --- |
| **Experimental Models: Organisms/Strains** |  |  |
| *C. elegans* N2 Bristol | CGC | N2 |
| *rde-1(ne219)* V; P*col-19*-RDE-1(*juIs346*) I | （Xu and Chisholm, 2011) | CZ14540 |
| P*col-19*-myr::mKate2(*zjuSi46*) IV | (Meng et al., 2020) | SHX364 |
| *rab-6.2(ok2254)* X outcrossed x2 | This paper | SHX932 |
| *Pcol-19*-PH::GFP(*zjuSi175*) I | This paper | SHX2000 |
| P*col-19*-GFP::RAB-1(*zjuSi253*) II | This paper | SHX2824 |
| P*col-19*-TGN-38::tagBFP(*zjuSi280*) X | This paper | SHX2994 |
| P*col-19*-GFP::RAB-1(*zjuSi253*) II; FRT-P*col-19*-myr::mKate2(*zjuSi46*) IV; P*col-19*-TGN-38::tagBFP(*zjuSi280*) X | This paper | SHX3017 |
| P*col-19*-PH::GFP(*zjuSi175*) I; P*col-19*-TGN-38::tagBFP(*zjuSi280*) X | This paper | SHX3031 |
| P*col-19*-GFP::MANS-2(*zjuSi302*) II | This paper | SHX3078 |
| P*col-19*-GFP::RAB-6.2(*zjuSi313*) I | This paper | SHX3151 |
| P*col-19*-PH::mKate2(*zjuSi321*) II | This paper | SHX3166 |
| P*col-19*-PH::GFP(*zjuSi175*) I ; P*col-19*-myr::mKate2(*zjuSi46*) IV | This paper | SHX3302 |
| P*ppk-1*-GFP(*zjuEx1756*) | This paper | SHX3303 |
| P*col-19*-mKate2::P4M(*zjuSi333*) V | This paper | SHX3336 |
| P*col-19*-PH::mKate2(*zjuSi321*) II; P*col-19*-GFP::PPK-1(*zjuEx1852*) | This paper | SHX3425 |
| P*col-19*-PH::mKate2(*zjuSi321*) II; P*col-19*-P4M::FKBP12(mTOR)::tagBFP, P*col-19*-GFP::FKBP1A(FKBP 2-108AA)::SAC1(2-517AA)(*zjuEx1859*) | This paper | SHX3432 |
| P*col-19*-PH::mKate2(*zjuSi321*) II; P*col-19*-GFP::FKBP1A(FKBP 2-108AA)::SAC1(2-517AA)(*zjuEx1865*) | This paper | SHX3446 |
| P*col-19*-PH::mKate2(*zjuSi321*) II ; P*col-19*-P4M::FKBP12(mTOR)::tagBFP(*zjuEx1867*) | This paper | SHX3448 |
| P*col-19*-PH::GFP(*zjuSi175*) I; P*col-19*-mKate2::P4M(*zjuSi333*) V | This paper | SHX3464 |
| P*col-19*-TGN-38::tagBFP(*zjuSi280*) X; P*col-19*-mKate2::P4M(*zjuSi333*) V | This paper | SHX3465 |
| *rde-1(ne219)* V; P*col-19*-PH::GFP(*zjuSi175*) I; P*col-19*-RDE-1(*juIs346*) I | This paper | SHX3761 |
| *rde-1(ne219)* V; P*col-19*-mKate2::P4M(*zjuSi333*) V; P*col-19*-RDE-1(j*uIs346*) I | This paper | SHX3762 |
| P*col-19*-GFP::MANS-2(*zjuSi302*) II; P*col-19*-TGN-38::tagBFP(*zjuSi280*) X, P*col-19*-mKate2::R12B2.2 (*zjuEx2075*) | This paper | SHX3806 |
| P*col-19*-mKate2::P4M(*zjuSi333*) V; P*col-19*-GFP::PPK-1(*zjuSi367*) X | This paper | SHX3870 |
| P*col-19*-PH::mKate2(*zjuSi321*) II;  P*col-19*-GFP::PPK-1(*zjuSi367*) X | This paper | SHX3871 |
| P*col-19*-mKate2::P4M(*zjuSi333*) V;  P*col-19*-GFP::PPK-1(c)(*zjuEx2142*) | This paper | SHX3901 |
| P*col-19*-myr::mKate2(*zjuSi46*) IV; P*col-19*-GFP::PPK-1(*zjuSi367*) X | This paper | SHX3918 |
| P*col-19*-mKate2::P4M(*zjuSi333*) V; P*col-19*-PPK-1(1-453)::GFP(*zjuEx2147*) | This paper | SHX3919 |
| *ppk-1(syb6134)* I*;* P*col-19*-PH::GFP(*zjuSi175*) I; P*col-19*-mKate2::P4M(*zjuSi333*) V | This paper | SHX3971 |
| *rab-6.2(ok2254)* X; P*col-19*-PH::GFP(*zjuSi175*) I; P*col-19*-mKate2::P4M(*zjuSi333*) V | This paper | SHX3972 |
| *rab-6.2(ok2254)* X; P*col-19*-TGN-38::tagBFP(*zjuSi280*) X; P*col-19*-mKate2::P4M(*zjuSi333*) V | This paper | SHX3994 |
| P*col-19*-mKate2::P4M(*zjuSi333*) V; P*col-19-*PPK-1(1-85)::GFP(*zjuEx2265*) | This paper | SHX4068 |
| P*col-19*-mKate2::P4M(*zjuSi333*) V; P*col-19-*PPK-1(84-457)::GFP(*zjuEx2267*) | This paper | SHX4070 |
| P*col-19*-GFP::MANS-2(*zjuSi302*) II; Pcol-19-mKate2::GBF-1(*zjuEx2292*) | This paper | SHX4114 |
| P*col-19*-PH::GFP(*zjuSi175*) I; Pcol-19-mKate2::GBF-1(*zjuEx2293*) | This paper | SHX4115 |
| P*col-19*-TGN-38::tagBFP(*zjuSi280*) X; Pcol-19-mKate2::GBF-1(*zjuEx2295*) | This paper | SHX4120 |
| P*col-19*-PH::mKate2(*zjuSi321*) II;  P*col-19*-TGN-38::FKBP12(mTOR)::tagBFP, P*col-19*-GFP::FKBP1A(FKBP 2-108AA)::SAC1(2-517AA)(*zjuEx2307*) | This paper | SHX4142 |
| P*col-19*-GFP::MANS-2(*zjuSi302*) II; P*col-19-*mKate2::ZC8.6 (*zjuEx2320*) | This paper | SHX4159 |
| P*col-19*-TGN-38::tagBFP(*zjuSi280*) X; P*col-19-*mKate2::ZC8.6(*zjuEx2330*) | This paper | SHX4169 |
| P*col-19*-myr::GFP(*zjuSi56*) IV;  P*col-19*-mKate2::P4M(*zjuSi333*) V | This paper | SHX4409 |
| *ppk-1(syb6134)* I | SunnyBiotech | PHX6134 |
