# Supplementary material for: Triggered Golgi membrane enrichment promotes PtdIns(4,5)P2 generation for plasma membrane repair
Source: J Cell Biol. 2023 May 9;222(8):e202303017. doi: 10.1083/jcb.202303017 (PMC10176212; doi:10.1083/jcb.202303017)
Supplement: Table S4 — lists primers used in this study. [file JCB_202303017_TableS4.docx]

**Table S4.** Primers used in this study

| **Oligonucleotides** | **Corresponding Plasmid No.** | **IDENTIFIER** |
| --- | --- | --- |
| CHR I seq SCI  cgtctctccacgatttacacactatttg | Chromosome I SCI sequencing | ZJU138 |
| CHR IV seq SCI  ggaagacccttagttccaaacaagtg | Chromosome IV SCI sequencing | ZJU236 |
| CHR II seq SCI  acgcccaggagaacacgttag | Chromosome II SCI sequencing | ZJU533 |
| accggtgagctctacctgtac | pSX3218 | ZJU1149 |
| RAB-6.2 ok2254 ex for  ctgcatatcgctacgtgg | *rab-6.2 KO* sequencing | ZJU1318 |
| RAB-6.2 ok2254 in for  tcaactttgggacaccgc | *rab-6.2 KO* sequencing | ZJU1319 |
| RAB-6.2 ok2254 rev  cagcgcaaggtaagtttagc | *rab-6.2 KO* sequencing | ZJU1320 |
| CHR V seq SCI  agcaaggttcttactacatgg | Chromosome V SCI sequencing | ZJU3750 |
| *ppk-1* inner for seq  aaatgctggagcatccgg | *ppk-1* mutation  sequencing | ZJU4811 |
| *ppk-1* ex test for 2#  ttcagataatggcttctcgg | *ppk-1* mutation  sequencing | ZJU4862 |
| *ppk-1* ex test rev 2#  ttgcagaggactctacag | *ppk-1* mutation  sequencing | ZJU4863 |
| pCR8-Pcol-19-X-GFP linker for  ggaggtggaggttcgggagg | pSX3217 | ZJU5028 |
| PPK-1 end for  tgacgctgtcatcagatcgcc | pSX3135 | ZJU5185 |
| PPK-1 453 rev  aactttttcggtcataaaagttaaaaatcgagatgcg | pSX3218 | ZJU5186 |
| PPK-1 C for  ttcaaaaagggaacagctctg | pSX3136 | ZJU5187 |
| PPK-1 453 GFP for  gcatctcgatttttaacttttatgaccgaaaaagttggaggtggaggttcgggaggtg | pSX3152 | ZJU5188 |
| PPK-1 83 rev  tacttttttgtacgaaactccaccctg | pSX3217 | ZJU5283 |
| PPK-1 (84-453) for  atgcccacgaatgctttgatgcaggc | pSX3218 | ZJU5284 |
| GFP mans-2 rev  gatgctcctgaggctcccgatgctccattagttaagactggaaccgggtg | pSX2530 | ZJU5395 |
| tgn-38 BFP for  ggaggacccttggagggtacaggatgaaattacgactcttcgtcgtgg | pSX2476 | ZJU5396 |
| tgn-38 BFP rev  ctgacgatgctcctgaggctcccgatgctccgtttccggcttcattttggcttagc | pSX2476 | ZJU5397 |
| GFP rab-1 for  catcgggagcctcaggagcatcggcagcaatgaaccctgaatacg | pSX2259 | ZJU5398 |
| GFP rab-1 rev  agatggcgatctgatgacagcgttaacaacatccaccgctcttcttg | pSX2259 | ZJU5399 |
| mKate2 R12B2.2 for  ggagggtacaggtagagctcaccggtatgtccgagctcatcaaggagaacatg | pSX3020 | ZJU5400 |
| mKate2 R12B2.2 rev  gtagatccgatgctcctgaggctcccgatgctccacggtgtccgagcttggatgggag | pSX3020 | ZJU5401 |
| mKate2 ZC8.6 for  ccgtggagcatcgggagcctcaggagcatcgatgaacagtggacatcccgactctg | pSX3266 | ZJU5611 |
| mKate2 ZC8.6 rev  gtcagaggcacgggcgcgagatgtcaccaccaacggaagaacggggctttc | pSX3266 | ZJU5612 |
